# Case Report: Diagnosis of bronchopulmonary candidiasis—refractory airway hyperresponsiveness, severe pneumonia, and normal serological markers

**DOI:** 10.3389/fmed.2025.1747382

**Published:** 2026-01-14

**Authors:** Dalong Zhang, Yuanjie Shang, Tongrui Zhang, Ying Liu, Xingguo Niu

**Affiliations:** Department of Critical Care Medicine, The Fifth Clinical Medical College of Henan University of Chinese Medicine (Zhengzhou People’s Hospital), Zhengzhou, Henan, China

**Keywords:** bronchitis, bronchopulmonary candidiasis, *Candida albicans*, diagnosis, pneumonia, treatment

## Abstract

**Background:**

Bronchopulmonary candidiasis is a clinically common type of pulmonary fungal disease, primarily caused by infection with Candida species (mostly *Candida albicans*). It typically manifests as a secondary infection when the body’s resistance is compromised. Clinically, bronchopulmonary candidiasis is primarily classified into three types: (1) bronchitic, (2) pneumonic, and (3) hypersensitivity.

**Methods:**

This paper reports two cases of bronchopulmonary candidiasis. The first case presented with bronchitis-type manifestations. Imaging studies showed no evidence of severe pneumonia, but the patient had persistent airway hyperresponsiveness that led to difficulty in weaning from mechanical ventilation. *Candida albicans* was identified in the bronchoalveolar lavage fluid (BALF), supporting a diagnosis of Candida bronchitis. The patient was successfully weaned following targeted antifungal therapy. The second case presented with the pneumonia variant, clinically manifesting as severe pneumonia. *Candida albicans* was also isolated from the BLAF. The patient recovered and was discharged after receiving combination antifungal therapy with caspofungin and isavuconazole.

**Results:**

Serological markers for fungal infection were normal in both patients, but pathogen cultures from BALF revealed *Candida albicans* growth. Combined with clinical presentations, these findings supported diagnoses of invasive Candida bronchitis and pneumonia, respectively.

**Conclusion:**

This report indicates that the clinical manifestations of bronchopulmonary candidiasis may be atypical relative to radiological and serological indicators. Although a single positive BALF culture is insufficient for definitive diagnosis, its integration with clinical characteristics can provide a crucial diagnostic clue. These cases thus provide valuable reference points for clinical diagnosis, though definitive validation through histopathological examination remains necessary.

## Introduction

Invasive pulmonary fungal infection (IPFI) refers to bronchopulmonary infections caused by fungal pathogens and accounts for approximately 15% of severe respiratory infections (1). Pathologically, fungal organisms invade the trachea, bronchi, and pulmonary tissues, leading to inflammation of the airway mucosa and the formation of pneumonic granulomas. In severe cases, this can progress to necrotizing pneumonia, which poses significant challenges for both clinical diagnosis and therapeutic management. Clinical studies have identified *Candida albicans* as the predominant fungal pathogen involved in respiratory infections (2). Clinically, *Candida albicans* is primarily associated with bronchopulmonary candidiasis, a common form of pulmonary fungal infection. This condition typically occurs as a secondary infection in immunocompromised individuals. The disease manifests in distinct clinical subtypes, requiring therapeutic strategies that address both the underlying predisposing factors and the fungal pathogen through antifungal treatment. Bronchopulmonary candidiasis is mainly classified into three types: (1) Candida tracheobronchitis (bronchitis type), (2) Candida pneumonia (pneumonia type), and (3) allergic bronchopulmonary candidiasis (hypersensitivity type) (3). The bronchitis subtype is defined by Candida infection confined to the bronchial mucosa, without extension into the lung parenchyma. This form represents the mildest variant among the three classifications and is frequently observed in patients with mildly impaired immune function or those who have recently undergone treatment with broad-spectrum antibiotics. Clinically, the presentation is predominantly limited to respiratory symptoms, such as cough and sputum production, with the sputum characteristically described as white mucus or frothy in appearance (4). In bronchitis-type cases, systemic symptoms are generally minimal, with body temperature typically remaining within the normal range of 36.0 °C–37.2 °C. A small proportion of patients may present with a low-grade fever, ranging from 37.3 °C to 38.0 °C. Pulmonary findings in these cases are either absent or mild, clinically mimicking chronic bronchitis. In contrast, the pneumonia-type form is characterized by parenchymal involvement and manifests with clinical features similar to acute pneumonia. This subtype arises when Candida species breach the bronchial mucosal defenses to invade the lung parenchyma, predominantly affecting individuals with severe immunosuppression, such as those experiencing neutropenia following chemotherapy, prolonged corticosteroid therapy, or organ transplantation. The disease course in these patients is typically rapid. Clinical manifestations primarily involve respiratory tract symptoms and systemic signs of infection, including fever, productive cough, chest pain, and dyspnea. In severe cases, the condition may progress to respiratory failure (5). The pneumonia type presents with characteristic inflammatory lung findings, including abnormal breath sounds over the affected areas. Some patients may also exhibit bronchial breath sounds and pleural friction rubs. This form progresses rapidly, worsening within days and potentially leading to pleural effusion, lung abscesses, and systemic candidiasis, often associated with a poor prognosis (6, 7). The allergic type, known as allergic bronchopulmonary candidiasis, is mediated by hypersensitivity reactions and primarily presents with allergic symptoms. This form does not involve direct fungal infection of lung tissue but rather an allergic response triggered by Candida antigens (e.g., hyphae, spores). It predominantly occurs in individuals allergic to Candida and is often associated with environmental exposure to Candida, such as damp environments or prolonged contact with moldy items (8). Allergic-type clinical manifestations primarily involve allergic respiratory symptoms accompanied by systemic allergic reactions, with bronchial asthma-like symptoms being the most characteristic presentation. This type does not involve pulmonary consolidation, and wheezing may be audible during episodes. Additionally, it is characterized by recurrent episodes that resolve after removal of triggers and anti-allergic treatment.

In recent years, the rapid advancement of modern medical technology, along with significant changes in clinical diagnostic and therapeutic methods, has contributed to an increased incidence of bronchopulmonary candidiasis (9). From a clinical perspective, the differential diagnosis of bronchopulmonary candidiasis remains a considerable challenge. Recent case studies indicate that relying solely on imaging studies is insufficient for an accurate diagnosis; additional investigations are necessary to confirm the condition (10). Diagnostic difficulties often impede timely and precise diagnosis and treatment, resulting in delayed intervention and a significantly worsened prognosis for some patients. Therefore, early and accurate diagnosis is essential for effective disease management and recovery.

This paper systematically examines the practical significance of diagnostic methods, such as imaging studies and pathogen identification, in diagnosing bronchopulmonary candidiasis through the analysis of two clinical cases. It aims to provide novel insights and methodologies for the clinical diagnosis of this condition.

## Case report

### Case 1: bronchitis form of bronchopulmonary candidiasis

#### Patient information

A 58-year-old female patient was admitted for worsening dyspnea lasting 2 days, occurring 34 days after cerebral hemorrhage surgery. Thirty-four days earlier, she experienced a sudden loss of consciousness accompanied by vomiting, with no apparent cause. A CT scan performed at another hospital revealed a left basal ganglia hemorrhage measuring approximately 60 ml. She underwent emergency burr hole drainage. Postoperatively, she received intracranial pressure reduction therapy, tracheostomy, and mechanical ventilation support. Although she could open her eyes spontaneously, she was unable to communicate verbally or move her limbs. During this period, the patient developed worsening dyspnea with wheezing and difficulty weaning from mechanical ventilation, prompting transfer to our Intensive Care Unit (ICU) for further management. The patient has a 12-year history of rheumatoid arthritis. Long-term oral anti-rheumatic medications include: chloroquine phosphate 0.25 g once daily; leflunomide 20 mg once daily; Total Glucosides of White Paeony Capsules 2 capsules three times daily; Alfacalcidol Tablets 2 tablets three times daily; And 8 years of hypertension, with peak blood pressure reaching 160/100 mmHg, controlled by regular medication; and a history of fractures in 2014 and 2022 (Table 1).

**TABLE 1:** Timeline of clinical events for the Case 1.

Time point	Clinical events	Investigations and findings
Day 0 (day of cerebral hemorrhage onset)	• Symptoms onset • Performed “trepanation and drainage” at an external hospital.	• CT scan of the head: Shows bleeding in the left basal ganglia region, approximately 60 ml.
Day 0∼day 33	• Postoperative management: Received intracranial pressure reduction, tracheostomy, and mechanical ventilation support. • Newly emerging issues: Developed dyspnea and wheezing, with difficulty weaning off mechanical ventilation.	• Not available
Day 34	• Transferred to ICU: Admitted due to worsening dyspnea and difficulty weaning from ventilator support. • Bronchoscopy: Revealed hyperemic airway mucosa with copious white, viscous secretions.	• Inflammatory markers: Mildly elevated, no significant abnormalities observed. • Imaging: Chest CT reveals diffuse inflammation in both lungs. • Pathogenology: Blood cultures, G test and GM test negative; Sputum fungal smear negative.
Day 35	• Diagnosis: BALF culture revealed *Candida albicans*. • Initiate targeted therapy: Begin antifungal treatment with Caspofungin.	• BALF culture: *Candida albicans* positive.
Days 35–65	• Nebulization, anti-inflammatory therapy, nutritional support • Manage complications	• Symptom improvement
Day 65	• Complete the antifungal treatment course	• Inflammatory markers decreased, oxygenation function improved.
Day 75 • (important clinical milestone)	• Successful extubation: Patient successfully weaned off mechanical ventilation and tracheal tube removed. • Repeat imaging studies and bronchoscopy procedures. • Transfer to rehabilitation department.	• CT scan shows resolution of bilateralpneumonia. • BALF did not detect *Candida albicans*, with decreased inflammatory markers and marked improvement.
Day 105	• Discharge to home	• Not available
Day 195	• Follow-up	• Conscious and alert, with no recurrence of infection.

BALF, bronchoalveolar lavage fluid.

#### Clinical findings

On admission, physical examination revealed a tracheostomy *in situ*, with the patient receiving mechanical ventilation in synchronized intermittent mandatory ventilation (SIMV) mode. Pulse oximetry showed an oxygen saturation of 98% on a fraction of inspired oxygen (FiO_2_) of 0.35. Vital signs were as follows: temperature, 36.6 °C; heart rate, 115 beats per minute; respiratory rate, 28 breaths per minute; and blood pressure, 162/75 mmHg. The Glasgow Coma Scale (GCS) score was 8 (E4V1M3). Pupils were bilaterally equal and round, approximately 4.0 mm in diameter, with sluggish light reflexes. Auscultation of the lungs revealed bilateral coarse breath sounds with expiratory wheezes. There was no spontaneous movement in the limbs, and muscle tone was increased; however, no pathological reflexes were elicited. Mild pitting edema was noted in both lower extremities.

**FIGURE 1:**
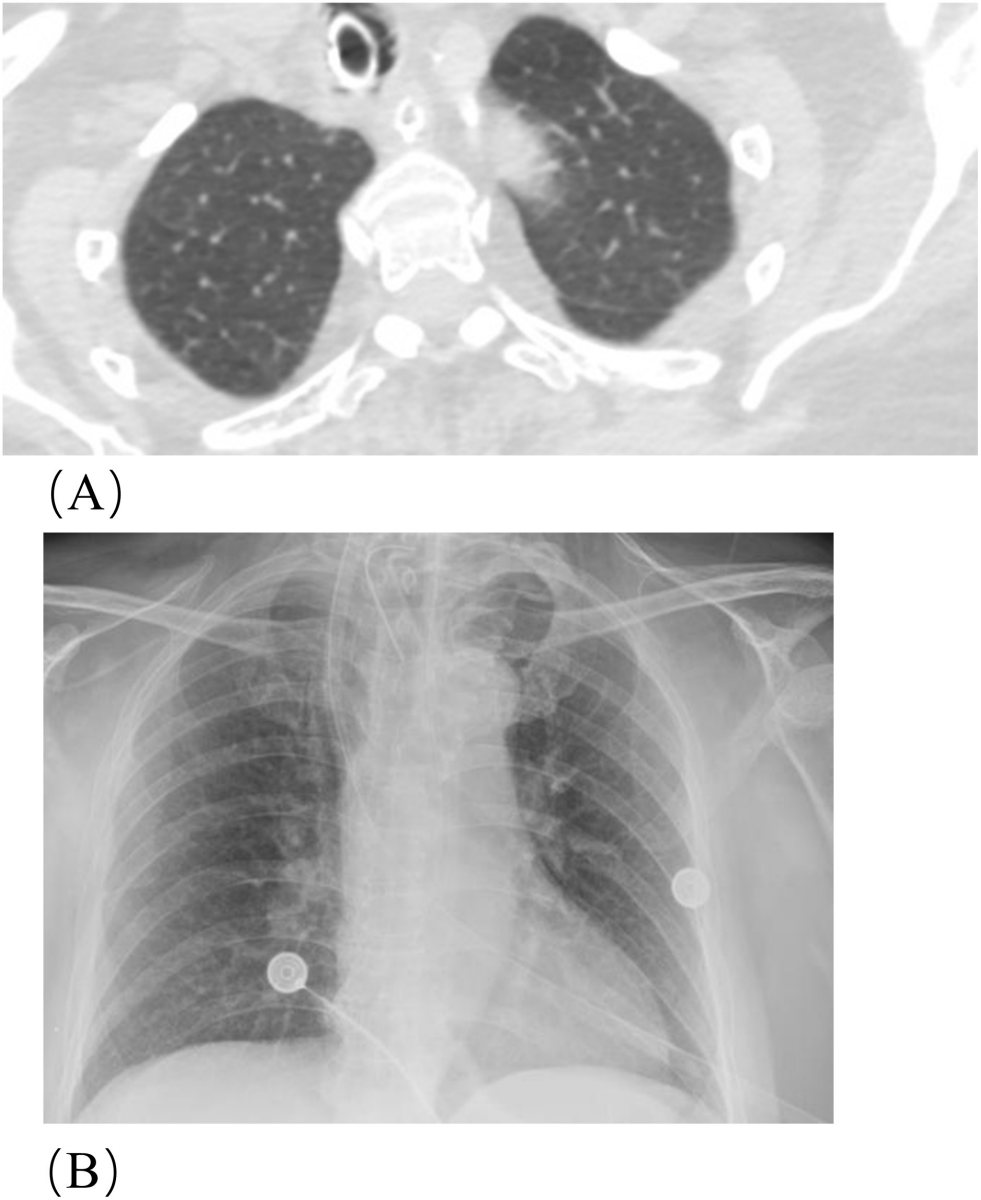
Chest CT and X-ray findings of Case 1 before treatment. **(A)** Scattered interstitial and congestive inflammatory changes are observed in left lung. **(B)** Chest X-ray reveals a symmetrical thoracic cage with regular pulmonary vascular patterns. No distinct areas of increased density are visible within the lung fields. The mediastinum is centrally located, with unremarkable hilar structures and a normal cardiac silhouette. Bilateral costophrenic angles are well defined.

**FIGURE 2:**
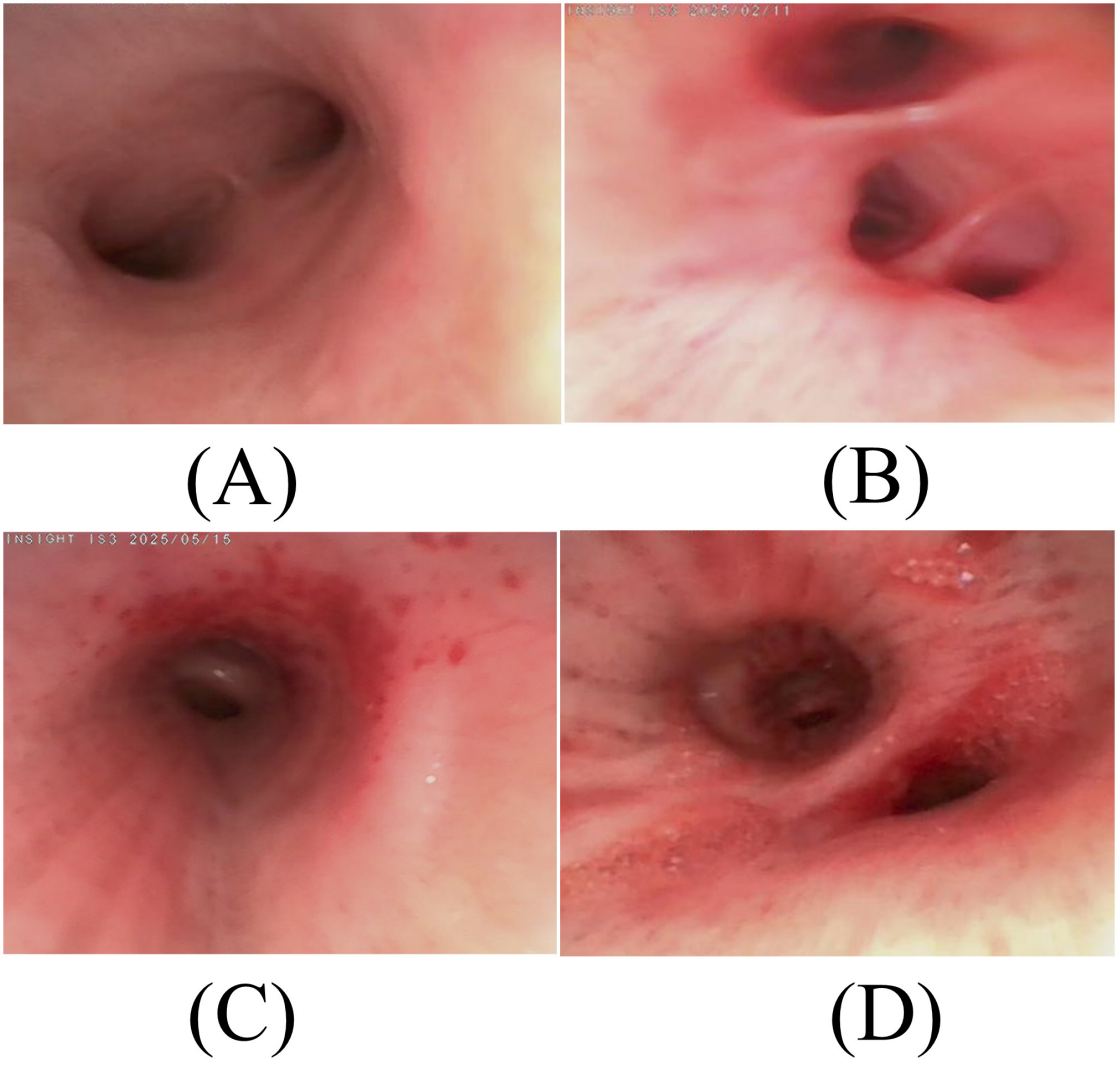
Bronchoscopic findings in Case 1 and Case 2 before treatment. **(A)** No significant narrowing or congestion/edema is visible in the airways. **(B)** Upon entering the bilateral main bronchi, the mucosa appears congested and edematous, with no narrowing or obstruction of the lumen. **(C)** The airway mucosa shows marked hyperemia and edema. **(D)** Diffuse white, patchy pseudomembranes are present on the bronchial mucosa, covered with purulent secretions, accompanied by mucosal hyperemia and edema.

Regarding auxiliary tests, inflammatory markers showed interleukin-6 (IL-6) at 1.9 pg/mL and tumor necrosis factor-alpha (TNF-α) at 1.8 pg/mL. Procalcitonin (PCT) was mildly elevated at 0.137 ng/mL, while high-sensitivity C-reactive protein (hs-CRP) measured 4.87 mg/L, within the relatively normal range. Arterial blood gas analysis revealed a pH of 7.481, pCO_2_ of 30.4 mmHg, lactate of 2.27 mmol/L, and an oxygenation index of 270 mmHg, indicating mild respiratory alkalosis and impaired oxygenation without significant hypoxemia. Coagulation studies showed a prolonged prothrombin time (PT) of 17.3 s, an elevated international normalized ratio (INR) of 1.46, and a mildly elevated D-dimer at 0.99 μg/mL. Notably, blood cultures for anaerobic and aerobic bacteria, along with (1,3)-β-D-glucan test (G test), and galactomannan antigen test (GM test), were negative, indicating no detectable bacterial or fungal pathogens in the blood. Fungal spore and hyphae smears showed no evidence of fungal elements. Imaging findings included chest computed tomography (CT), which revealed scattered interstitial and congestive inflammation in both lungs with bilateral pleural effusions but no definite consolidation or infiltrates (Figure 1A). Chest X-ray showed a symmetrical thoracic cage with regular pulmonary vascular patterns and no abnormal densities in the lung fields, inconsistent with typical severe pneumonia (Figure 1B). To further identify the pathogen, bronchoscopy revealed hyperemic airway mucosa with copious white viscous secretions (Figures 2A, B). Bronchoalveolar lavage fluid (BALF) culture indicated *Candida albicans* infection. Immunological assessment showed decreased CD4^+^ T-cell count at 201.47/μL and CD8^+^ T-cell count at 96.4/μL, suggesting immunosuppression.

#### Diagnosis

Based on imaging studies, blood gas analysis, and clinical presentation, the difficulty in weaning from mechanical ventilation is primarily attributed to *Candida albicans* bronchitis rather than severe pneumonia.

##### Differential diagnosis

Allergic bronchopulmonary aspergillosis (ABPA): ABPA is an hypersensitivity reaction commonly seen in patients with asthma or cystic fibrosis. Chest X-ray/CT reveals migratory pulmonary infiltrates, with marked elevation of eosinophils in blood counts and significantly increased total IgE levels, while inflammatory markers are typically normal. In this case, imaging studies showed no pulmonary infiltrates, and the patient presented with immunosuppression but no history of asthma or cystic fibrosis.

#### Interventions

Therapeutically, the patient remained on ventilator support (SIMV mode) post-admission, requiring a low oxygen concentration (35%) and maintaining normal pulse oximetry. Multiple weaning attempts failed due to airway hyperresponsiveness, although clinical symptoms remained mild, with no fever, purulent sputum, or significant hypoxemia indicative of severe pneumonia. Subsequent adjustments included targeted antimicrobial therapy with caspofungin (First dose: 70 mg, followed by 50 mg daily for 25 days.) for infection control, supplemented by comprehensive treatment comprising nebulization, anti-inflammatory agents, nutritional support, and rehabilitation exercises. During this period, feeding intolerance developed but gradually improved after adjusting the enteral nutrition plan and adding gastrointestinal motility agents. Throughout treatment, inflammatory markers remained stable, oxygenation indices showed minimal fluctuation, and consciousness improved (GCS score increased to 10). Seventy-five days postoperatively, the patient successfully weaned off mechanical ventilation and underwent tracheostomy tube removal, with some recovery of neurological function. Repeat imaging studies revealed CT scans showing resolution of scattered interstitial and obstructive inflammation in both lungs, with no abnormal findings. BALF testing detected no *Candida albicans*, and inflammatory markers decreased, indicating significant improvement.

#### Follow-up

The patient was subsequently transferred to the Rehabilitation Department for continued treatment and discharged 1 month later. At the 3-month follow-up after discharge, the patient was alert and oriented, able to eat orally, and capable of performing bedside activities and walking indoors. There were no recurrences of infection, seizures, or rebleeding.

### Case 2: pneumonic form of bronchopulmonary candidiasis

#### Patient information

A 67-year-old male patient was admitted for “dyspnea for 7 days, worsening over the past half day.” The patient developed dyspnea 7 days ago following strenuous farm labor, with slight relief after rest. He exhibited signs of fever, and symptomatic treatment with medications from a local clinic proved ineffective (previous interventions required: Piperacillin Sodium and Tazobactam Sodium). Symptoms significantly worsened one day prior, leading to referral from another hospital to our institution. He was admitted to the ICU through the emergency department with a diagnosis of severe pneumonia and respiratory failure. The patient had no significant past medical history (Table 2).

**TABLE 2:** Timeline of clinical events for the Case 2.

Time point	Clinical events	Investigations and findings
Day-7	• Experiencing shortness of breath after strenuous farm work.	• Not available
Day-6 to -2	• Symptomatic treatment was administered at a local clinic, but the results were unsatisfactory.	• Not available
Day-1	• The condition worsened and Transferred from another hospital to this hospital.	• Not available
Day 0 admission date	• Admission diagnosis: Diagnosed with severe pneumonia and respiratory failure. Emergency treatment: Immediately initiate NIV and empirical antimicrobial therapy. Perform bronchoscopy and bronchoalveolar lavage.	• Imaging studies reveal signs of severe infection in both lungs. Laboratory tests: No significant elevation in inflammatory markers; low oxygenation index indicating acute respiratory failure. Pathogenology: Blood cultures, G test and GM test negative; Sputum fungal smear negative.
Day 0–day 1	• Pathogen confirmation: mNGS of bronchoalveolar lavage fluid detected *Candida albicans*. Revised diagnosis: Based on clinical and pathogen findings, the diagnosis is *Candida albicans* pneumonia.	• Pathogen: BALF mNGS results positive for *Candida albicans*. Clinical assessment: Persistent severe hypoxemia with markedly elevated D-dimer levels, clinically suspected pulmonary embolism.
Day 2–day 4	• Caspofungin antifungal therapy	• Poor therapeutic response; consider combination therapy.
Day 5–day 35	• Change in treatment regimen: Caspofungin combined with Isavuconazole for antifungal therapy.	• Continuously monitor clinical symptoms, oxygenation indices, and inflammatory markers.
Day 36	• Treatment completed: Antifungal therapy concluded	• Improvement in patients’ oxygenation index and inflammatory markers.
Day 40	• Discharge from the hospital	• The patient’s condition is stable and meets the criteria for discharge.
Day 130	• Follow-up	• Follow-up confirmed no complications and good recovery.

NIV, non-invasive ventilation; mNGS, metagenomic next-generation sequencing.

#### Clinical findings

**FIGURE 3:**
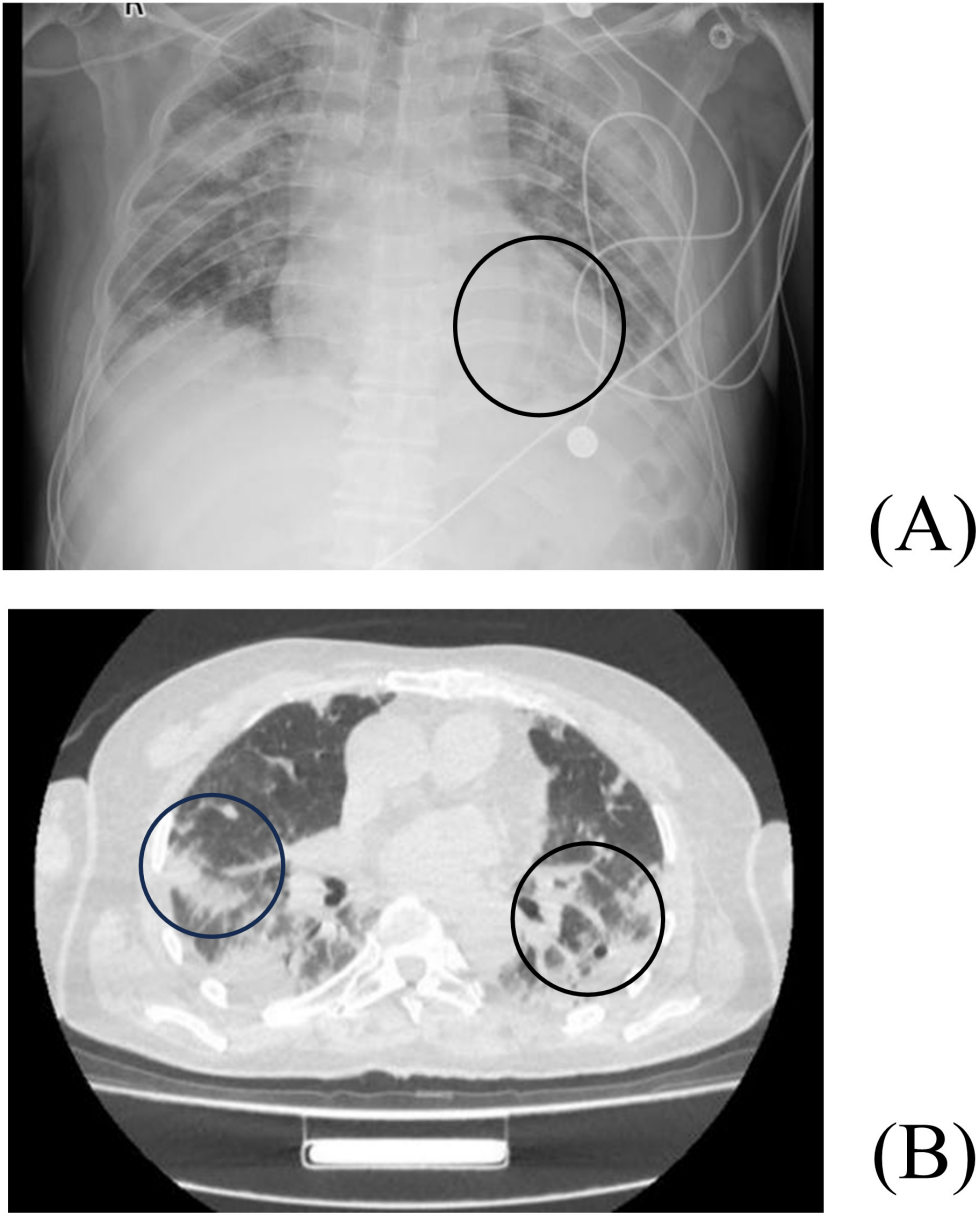
Chest X-ray and CT Findings in Case 2 before treatment. **(A)** X-ray examination reveals increased and blurred pulmonary markings bilaterally. Patchy areas of increased density with indistinct margins are visible in both lungs. The cardiac silhouette is normal in size, and the costophrenic angles are obscured bilaterally. **(B)** CT scan reveals multiple inflammatory lesions in both lungs.

On admission, physical examination revealed a temperature of 36.4 °C (97.5°F), heart rate of 62 bpm, respiratory rate of 28 breaths per minute, and blood pressure of 135/68 mmHg. Coarse breath sounds were noted bilaterally, accompanied by audible wheezes and moist rales in the bilateral lower lung fields. The heart rhythm was regular without murmurs. Supportive tests included a chest X-ray showing patchy bilateral pulmonary opacities with blurred margins (Figure 3A). CT further confirmed multiple bilateral pulmonary infiltrates with minimal pleural effusion, consistent with severe pulmonary infection (Figure 3B). Inflammatory markers revealed a markedly elevated hs-CRP level of 48.23 mg/L. Twelve infection-related immunological factors remained within normal ranges. Procalcitonin was slightly elevated at 0.137 ng/mL, consistent with the presence of an inflammatory process. D-dimer was elevated at 9.03 μg/mL, and fibrin degradation products (FDP) measured 35.85 μg/mL, indicating secondary hyperfibrinolysis and potential thrombotic risk. Arterial blood gas analysis under 60% oxygen supplementation showed an oxygenation index of only 132 mmHg, accompanied by hypoxemia (pO_2_ 79.5 mmHg) and lactate of 1.32 mmol/L, consistent with acute respiratory failure. Brain natriuretic peptide (BNP) was significantly elevated at 750.3 pg/mL, suggesting cardiac involvement. Immunological testing indicated a low CD4^+^ T-cell count (421.9/μL), potentially reflecting immunosuppression. Hepatic and renal function were largely normal, although albumin levels were decreased (28.1 g/L), suggesting malnutrition. Notably, blood cultures for both anaerobic and aerobic bacteria, along with G test and GM test were all negative, indicating insufficient evidence of systemic fungal infection. To confirm the pathogen diagnosis, bronchoscopy was performed (Figures 2C, D), and BALF was collected for Metagenomic Next-generation Sequencing (mNGS). Results revealed the presence of *Candida albicans* in the lavage fluid.

#### Diagnosis

Based on the patient’s clinical presentation, imaging findings, and microbiological results, although systemic fungal infection markers were mild, the detection of *Candida albicans* in BLAF, combined with the disease course, severe respiratory symptoms, and radiographic findings, led to a preliminary diagnosis of *Candida albicans* pneumonia.

##### Differential diagnosis

Pulmonary tuberculosis: Often presents with systemic toxic symptoms such as afternoon low-grade fever, night sweats, fatigue, weight loss, insomnia, and palpitations. Radiographic lesions typically occur in the apical regions or supraclavicular areas of the lungs, exhibiting heterogeneous density that resolves slowly and may form cavities or spread within the lungs. Mycobacterium tuberculosis can be detected in sputum. However, no Mycobacterium tuberculosis was found in the patient’s sputum in this case.

Bacterial pneumonia: Patients present with fever, cough, sputum production, chest pain, and dyspnea. Total white blood cell count is markedly elevated, with increased C-reactive protein and calcitonin levels. Chest X-ray or CT shows patchy shadows or consolidation in the lungs. Significant improvement occurs with antibiotics targeting Gram-negative or Gram-positive bacteria. This patient received antibiotic therapy prior to admission, but symptoms did not improve.

Simple *Candida albicans* colonization: No specific symptoms, normal inflammatory markers, and no evidence of tissue invasion. When considering bacterial pneumonia complicated by *Candida albicans* colonization, *Candida albicans* may be detected in the patient’s BLAF, and antibiotic treatment for bacterial pneumonia yields marked improvement.

#### Interventions

Upon admission, the patient immediately received non-invasive ventilatory support and empirical antimicrobial therapy, with sputum culture and pathogen testing initiated. The patient initially received piperacillin sodium and tazobactam sodium (4.5 g every 8 h, three times daily) for treatment against Gram-negative and Gram-positive bacteria, but symptoms did not show significant improvement. Targeted antifungal therapy was subsequently initiated. Caspofungin (initial dose 70 mg, followed by 50 mg daily) showed some improvement but remained suboptimal. Suspecting severe fungal infection, isavaconazole was added after 3 days (200 mg every 8 h for the first six doses, then 200 mg once daily). After 1 month of combined therapy, the patient’s condition improved. Through aggressive antimicrobial treatment, respiratory support, nutritional intervention, and symptomatic management, the patient’s oxygenation improved modestly.

#### Follow-up

After a 40-day hospitalization, the patient was discharged to recover at home. At the 3-month follow-up, the patient had fully recovered and could completely discontinue oxygen therapy. Dyspnea symptoms had significantly improved to resolution. The patient exhibited good mental status, appetite, and sleep, with stable vital signs. Pulmonary imaging showed marked absorption of lesions, with a significant reduction in the size of the original consolidation foci. Pulmonary function had markedly improved compared to the acute phase. There was no evidence of recurrent infection.

#### Patient perspective

The patient’s family reported a history of farming activities prior to symptom onset and expressed concern about a possible environmental link to the illness. This suggests the need for enhanced immunity going forward, while also emphasizing the importance of protective measures during farming to interrupt transmission routes and safeguard susceptible individuals.

## Discussion

The bronchitis type of bronchopulmonary candidiasis presents as a relatively mild form of the disease, primarily characterized by Candida colonization and infection of the bronchial mucosa. This triggers localized inflammation without extensive invasion of the lung parenchyma. Its clinical presentation resembles chronic bronchitis and includes symptoms such as cough, sputum production (typically white, viscous, or stringy), dyspnea, wheezing, and airway hyperresponsiveness. Systemic toxic symptoms (e.g., high fever, chills) are usually absent or mild. Chest CT may reveal non-specific signs of airway inflammation, such as bronchial wall thickening, a tree-in-bud pattern, or mosaic perfusion (11). While these findings suggest bronchial inflammatory changes, they cannot distinguish fungal infection from other etiologies. Laboratory diagnosis also has limitations. Since *Candida albicans* commonly colonizes the oropharynx, sputum culture results are prone to contamination or colonization interference, leading to a high false-positive rate. Although the G test and GM test—auxiliary diagnostic tools for systemic fungal infections (12)—were negative in both cases, suggesting the absence of systemic invasive infection, these tests cannot be relied upon to establish a definitive diagnosis. Case 1 primarily presented with airway hyperresponsiveness and weaning difficulties, initially suspected to have severe pneumonia. However, the imaging findings did not align with typical characteristics of severe pneumonia, leading to exclusion of this diagnosis. To further clarify the etiology, bronchoscopy revealed extensive mucosal hyperemia with copious white viscous secretions, providing crucial morphological evidence of localized fungal infection. Subsequent pathogen culture of BLAF confirmed *Candida albicans* growth. Integrating the patient’s clinical presentation, bronchoscopic findings, and microbiological evidence, the diagnosis was finalized as *Candida albicans* bronchitis, identified as the primary cause of weaning difficulties. Antibiotic therapy targeting the concomitant bacterial infection was initiated, supplemented with specific antifungal agents based on susceptibility testing. Following comprehensive treatment, pulmonary inflammation was effectively controlled, oxygenation significantly improved, and the patient was successfully weaned off mechanical ventilation.

Case 1’s most prominent clinical manifestations were worsening dyspnea with wheezing and difficulty weaning from mechanical ventilation, reflecting the airway hyperresponsiveness characteristic of Candida tracheobronchitis. Candida, acting as an allergen and inflammatory stimulus within the airways, triggered bronchospasm, mucosal edema, and increased secretions. This led to heightened airway resistance, manifesting as wheezing and ventilator dependence. Although sputum culture, blood culture, G test, and GM test were all negative, empirical antifungal therapy based on BLAF analysis improved the patient’s condition. Case 1 presented atypical symptoms compared to typical candidal bronchitis, characterized by more persistent symptoms and a prolonged course. Potential contributing factors include: (1) the patient’s underlying medical history, comprising the postoperative status following massive cerebral hemorrhage, which resulted in a hypercatabolic and stressed condition; and (2) long-term immunosuppression, associated with a 12-year history of rheumatoid arthritis requiring long-term oral anti-rheumatic medications, including immunosuppressants.

Pneumonic form of bronchopulmonary candidiasis, commonly known as “Candida pneumonia,” refers to a condition in which Candida not only affects the bronchi but also invades the lung parenchyma, causing inflammatory lesions. This form progresses rapidly and is prone to complications. Clinical symptoms primarily include high fever and severe coughing; in severe cases, chest pain and dyspnea may occur. Case 2 presented with an acute exacerbation of dyspnea and respiratory failure. Physical examination revealed bilateral wheezing and moist rales, with a low oxygenation index of 132 mmHg, consistent with the diagnosis of “severe pneumonia and acute respiratory failure.” Regarding imaging findings, the most notable radiographic feature of Candida pneumonia is the “absence of specific signs.” It may present with patchy opacities, ground-glass opacities, consolidation, or minimal pleural effusion (13). However, these manifestations are common to various pulmonary diseases. Therefore, the imaging findings in Case 2 only confirm severe pneumonia and cannot definitively diagnose Candida infection (14). Meanwhile, blood and sputum culture results are susceptible to interference from colonization, leading to false positives or false negatives (15). Furthermore, the patient’s G-test and GM-test were negative, which added to the diagnostic challenge. Subsequently, bronchoscopy was performed, and BALF was collected for mNGS. The analysis detected *Enterococcus faecium*, *Klebsiella aerogenes*, *Klebsiella pneumoniae*, and *Candida albicans*. However, given that *Candida albicans* commonly colonizes the upper respiratory tract and the absence of histopathological evidence, a definitive diagnosis of invasive infection could not be established. Nevertheless, the following clinical clues were considered: (1) a clear history of exposure to a moldy environment; (2) lack of clinical improvement despite adequate prior broad-spectrum antibacterial therapy both in and out of the hospital; and (3) a prominent number of *Candida albicans* sequences in the mNGS report. Therefore, *Candida albicans* was highly suspected as a potential causative pathogen. Based on this suspicion, antifungal therapy was initiated with caspofungin on the second hospital day (loading dose 70 mg, followed by 50 mg daily). Due to a poor response after 3 days, isavuconazole was added (200 mg every 8 h for the first 48 h, then 200 mg once daily). Following 1 month of combination therapy, the patient’s condition improved. This therapeutic response further supports the clinical impression of Candida pneumonia rather than typical bacterial pneumonia.

In summary, these two cases provide valuable educational insights for the diagnosis and management of pulmonary *Candida albicans* disease. According to the IDSA guidelines on Candida infections, recommendations for treating Candida in respiratory specimens explicitly state that Candida growth in respiratory secretions typically indicates colonization and rarely necessitates antifungal therapy. The core distinction between colonization and infection lies in whether microorganisms invade host tissues and trigger pathological reactions, with histopathological evidence serving as the gold standard for diagnosis. We recognize that relying solely on positive BALF cultures without histopathological confirmation is insufficient for diagnosing invasive pulmonary candidiasis. However, in these two patients, given the absence of other clear pathogen explanations in clinical presentation and imaging, the detection of *Candida albicans* in bronchoalveolar lavage fluid, and the significant improvement following targeted antifungal therapy, we speculate that *Candida albicans* may have played a pathogenic role in this specific context rather than merely being a colonizing organism. Therefore, clinicians diagnosing invasive *Candida albicans* infections must comprehensively evaluate all test results. Empirical treatment may be initiated promptly to improve early detection and cure rates for these infections. Normal serological markers cannot rule out bronchopulmonary candidiasis. For patients with specific clinical presentations, combined evaluation of BALF culture and treatment response provides valuable clinical decision-making guidance for early empirical intervention in bronchopulmonary candidiasis.

On the other hand, this case report also has certain limitations, such as a small number of cases and limited follow-up time, making it unclear how patients’ conditions progressed and recovered after discharge. Further in-depth research is anticipated in the future.

## Data Availability

The original contributions presented in this study are included in this article/supplementary material, further inquiries can be directed to the corresponding author.
